# Graphene Growth on Electroformed Copper Substrates by Atmospheric Pressure CVD

**DOI:** 10.3390/ma15041572

**Published:** 2022-02-19

**Authors:** Lorenzo Pedrazzetti, Eugenio Gibertini, Fabio Bizzoni, Valeria Russo, Andrea Lucotti, Luca Nobili, Luca Magagnin

**Affiliations:** 1 Department of Chemistry, Materials and Chemical Engineering “Giulio Natta”, Politecnico di Milano, 20131 Milano, Italy; lorenzo.hnd@gmail.com (L.P.); eugenio.gibertini@polimi.it (E.G.); fabiobizzoni@gmail.com (F.B.); andrea.lucotti@polimi.it (A.L.); luca.magagnin@polimi.it (L.M.); 2 Energy Department, Politecnico di Milano, 20133 Milano, Italy; valeria.russo@polimi.it

**Keywords:** graphene, CVD, electroforming

## Abstract

Chemical vapor deposition (CVD) is regarded as the most promising technique for the mass production of graphene. CVD synthesis under vacuum is the most employed process, because the slower kinetics give better control on the graphene quality, but the requirement for high-vacuum equipment heavily affects the overall energy cost. In this work, we explore the possibility of using electroformed Cu substrate as a catalyst for atmospheric-pressure graphene growth. Electrochemical processes can produce high purity, freestanding metallic films, avoiding the surface defects that characterize the rolled foils. It was found that the growth mode of graphene on the electroformed catalyst was related to the surface morphology, which, in turn, was affected by the preliminary treatment of the substrate material. Suitable conditions for growing single layer graphene were identified.

## 1. Introduction

Since its first synthesis, graphene has been a hot topic for many research groups [1], because of its novel properties, intimately linked to its zero-gap semiconductor quality [2]. It is known that chemical vapor deposition on transition metal catalysts is one of the most promising methods for growing large areas of poly and monocrystalline graphene, and copper is one of the best substrates to do so [3,4]. Low pressure processes are already thoroughly characterized and are known to yield very good results in term of quality and domains size [5,6]. Atmospheric pressure CVD (AP-CVD), on the other hand, is less widespread, because of the substantially different kinetics involved [7] and, hence, the inherent difficulties in producing homogeneous graphene layers on wafer scale [8]. In mixed atmospheres of hydrogen and methane, the growth pressure was found to dictate the number of layers in the graphene material [9]. In particular, self-limiting growth of monolayer graphene on copper foil was observed only at low pressures (below 10 Torr), while the number of graphene layers increased as the growth pressure rose, so that multilayer graphene formed at a pressure of 120 Torr.

Alternatively, the H_2_/CH_4_ gas mixture can be diluted with an inert gas such as argon; two different growth modes have been identified in these atmospheres [10]. The first mode is associated with higher methane content in the gas mixture and consists in the growth of graphene flakes over the initial graphene layer covering the Cu substrate. In the second mode, occurring at lower methane content, few-layer graphene flakes form, where subsequent layers grow between the original ones and the substrate.

Along with these limitations, synthesis performed at atmospheric pressure has the advantage of excluding high vacuum equipment and reducing largely the production costs, which heavily affect CVD graphene mass production and pose a high barrier to its technological breakthrough [11]. The aim of the presented work was to characterize the AP-CVD graphene growth, using fully self-developed catalytic Cu substrates. To do so, electroforming from a commercial solution was employed, followed by delamination and post treatments of the free-standing metal foils. Electroforming has developed significantly over the last few decades, thanks to progresses in material science and manufacturing technologies; the advantages in employing such a technique are mainly related to its feature of atom-by-atom process, which allows for controlling catalysts structure and morphology [12], a fundamental task in CVD graphene synthesis [13]. In addition, this process can avoid the formation of surface defects, such as scratches and carbon contamination, commonly observed in commercial Cu foils as a result of the rolling operation [14,15].

The quality of the prepared materials was assessed by different characterization techniques: X-Ray fluorescence (XRF), X-Ray diffraction (XRD), and atomic force microscopy (AFM) were used to evaluate substrate purity, crystalline orientation and surface roughness. After electroforming, Cu foils underwent annealing [16] in hydrogen flow and electropolishing [17]. Characterization was repeated on the treated samples to identify the morphology parameters to be related to the quality of graphene films [18]. To understand how system-specific features affect graphene nucleation and growth, different processes were designed and tested on as-plated Cu, annealed Cu, and annealed-polished Cu. Raman spectroscopy relies on the inelastic scattering of coherent light to probe materials properties, especially when the symmetry of the probed system is well known. It is especially valuable to investigate and to assess graphene properties; atomically, thin carbon films show very marked fingerprints when exposed to monochromatic light, and a precise analysis and interpretation of these features provides an effective description of the system under analysis [19].

## 2. Materials and Methods

### 2.1. Preparation of Materials

**Figure 1:**
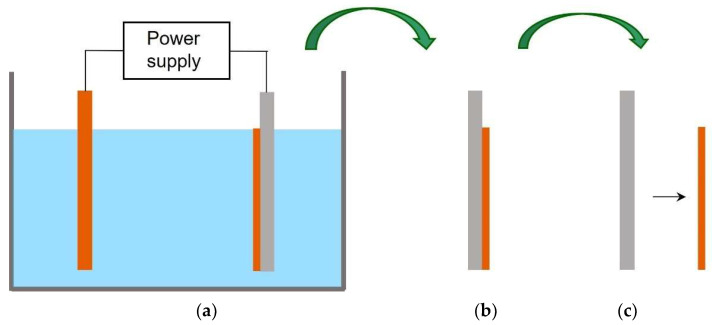
Preparation of Cu substrates by electroforming: (**a**) electrolytic cell, (**b**) plated mandrel, (**c**) free-standing Cu foil.

Electroformed substrates were prepared by using a stainless-steel mandrel as the cathode and a Cu plate as the sacrificial anode. The cell was set up in the standard two electrodes configuration; the electrolyte used was the Cuproplus bath for Cu plating, supplied by Tecnochimica; plating parameters were used according to the product datasheet. Resulting free-standing Cu foils were 2 cm × 2 cm in size, with a thickness of 50 µm and a good surface finishing. The setup for preparing the Cu foils is illustrated in Figure 1. Electropolishing electrolyte was made by 25% *v*/*v* phosphoric acid, 25% *v*/*v* ethanol, 5% *v*/*v* 2-propanol, and 0.1 M urea [13]; the process was carried out by applying 300 mA/cm^2^ current density for 30 s. Both substrate annealing and graphene chemical vapor deposition were performed in a tubular furnace using a quartz vial; both treatments were carried out at 1000 °C and atmospheric pressure. In the annealing treatment, pure hydrogen was supplied to the vial at the flow rate of 5 NL/h for two hours, while the CVD atmosphere was a H_2_-CH_4_ gas mixture, with CH_4_ content of 3 vol%. Further experimental details are reported elsewhere [20]. To investigate which were the best conditions for graphene growth, surface finishing of Cu foils and CVD reaction time were changed. As-plated Cu foils were exposed to the CH_4_/H_2_ flow for 2, 4, 6 and 8 min, while the annealed and the annealed-polished substrates were subjected to process times ranging from 2 to 20 min.

### 2.2. Characterization Methods

Purity of the obtained samples was assessed by a Fischerscope X-ray Xan instrument, while surface morphology of Cu foils was investigated by using a NT-DMT SOLVER PRO atomic force microscope, with a scanning area of 100 µm^2^ and a vertical resolution of 0.1 nm. XRD patterns were acquired by means of a Philips PW1830 spectrometer, on a surface area of 2 cm^2^. Scanning electron microscopy (SEM) analysis was performed using a ZEISS EVO 50 EP instrument equipped with a field-emission gun, under the accelerating potential of 5 kV. Raman spectra were acquired by means of a Horiba LabRam HR800 spectrometer equipped with an argon ion laser operating at 514 nm (2.41 eV). The scattered radiation was collected through a 50 × objective keeping the beam power below 1 mW to avoid sample heating and graphene damage. The intensity correction system (ICS) was always kept active, and a diffraction grating with 600 lines/mm was used; acquisition time was always 45 s.

## 3. Results

**Figure 2:**
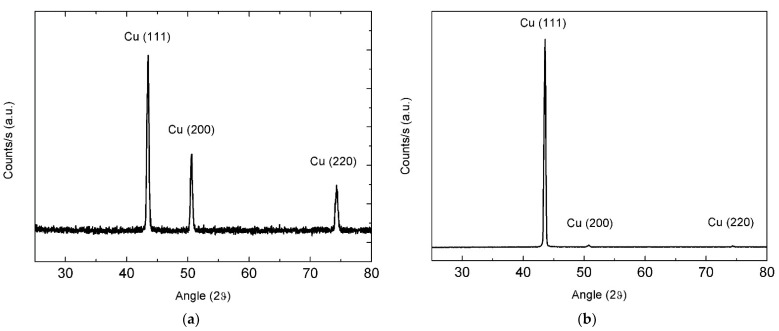
XRD patterns of the copper foil before (**a**) and after hydrogen annealing (**b**).

Graphene catalytic growth on Cu is generally considered as a surface deposition process, with a typical adsorption-aggregation mechanism [21]. From this point of view, presented work aims to study nucleation and growth of graphene and few-layer graphene (FLG) on a Cu substrate fully self-produced employing metal electroforming. In addition to Cu, no impurity elements were detected by XRF in these substrates. XRD patterns for pristine Cu foils showed an intense (111) peak together with (200) and (220) reflections. After annealing, almost all the crystallites exhibited (111) orientation (Figure 2a,b). Grain growth promoted by the annealing treatment is expected to favor the competitive expansion of crystals that expose close-packed planes with lower surface energy, leading to the observed preferential orientation. This was highly beneficial for growing graphene, because of the small mismatch at Cu/G interface on (111) crystal planes [22]. To evaluate the effect of a smoother surface, electropolishing was also carried out on annealed Cu foils (see Section 2.2). Measured root-mean-square roughness was found to be reduced from 55 nm (as-plated copper foil) to 4.1 nm for the annealed copper foil and to 3.8 nm for the annealed-polished copper foil.

**Figure 3:**
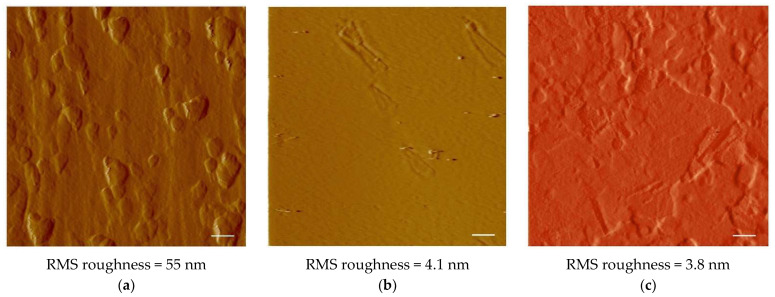
AFM images of the different substrates: (**a**) as-plated Cu, (**b**) annealed Cu, and (**c**) annealed-polished Cu. The scale bars are 1 μm.

Surface morphology of the as-plated foil is characterized by several protrusions roughly globular in shape; somewhere, elongated terraces are also observed (Figure 3a).

In the annealing treatment, surface and volume diffusion can produce atomic redistribution, causing the observed reduction in surface roughness. Actually, the annealed surface looks highly smooth, with rare protrusions probably deriving from original large globules flattened only partially during the annealing treatment (Figure 3b).

The electropolished surface appears more heterogeneous and consists of terraces and depressions with different sizes delimited by shallow steps (Figure 3c). Electropolishing converted the original annealed surface, substantially smooth with few protrusions, into a less uniform surface, characterized by a small vertical span in the roughness profile. The result is a minimal decrease of surface roughness, from 4.1 nm to 3.8 nm.

Raman spectrum of graphene layers features several bands, three of which are of uttermost importance when it comes to describing the main properties of the system of interest. The G band is located, in pristine samples, at 1582 cm^−1^; it is the only first-order Raman process in graphene and is associated to the degenerate E_2g_ phonon at the Γ point of the Brillouin zone. At 1350 cm^−1^ the D band is usually found, where “D” stands for “defect”: in fact, D Raman peak is originated by a second order process that involves a phonon at the Brillouin zone corner (A_1g_) and a defected state, treated as a 0 frequency quasi-particle to preserve the formalism [19,23,24]. Finally, at 2690 cm^−1^, the 2D band is observed; the 2D peak is, indeed, a second-order process where two phonons with opposite wave vectors were involved; hence, the momentum conservation is satisfied without the need of a defect state [25]. The 2D band description is of critical importance when analyzing the Raman spectrum of graphene films: its shape, full width at half maximum (FWHM), and intensity ratio with the G band (which remains unchanged with respect to film order and number of layers) are the necessary information needed to describe the probed nanomaterial. For monolayer graphene, an intensity ratio between G and 2D peaks (I_G_/I_2D_) is accepted to be lower than 1, and 2D Raman line shape has a FWHM of 24 cm^−1^ and is fitted by a single component Lorentzian function [26]. The spectrum transition to FLG must be carefully treated for Bernal stacked layers and for turbostratic, or rotationally faulted, layers. In the first scenario, the 2D band is usually fitted by a number of Lorentzian functions proportional to the number of layers, up to five [23]. The lack of a stacking order in FLG, on the other side, generates a broader 2D line shape (50 cm^−1^), which is usually fitted by a single Lorentzian function, as in the case of single layer graphene (SLG). This is due to the decoupling of the layers comprising the turbostratic FLG, each of which retains a high percentage of the SLG transport properties [27]. In the latter case, a complete description of the Raman features is needed to estimate the overall number of layer merged in the film [28]: position and linewidth of the 2D band and I_2D_/I_G_ intensity ratio.

### 3.1. As-Plated Cu Substrate

**Figure 4:**
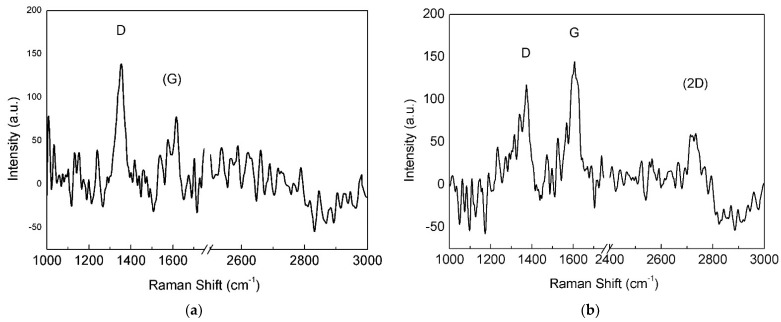

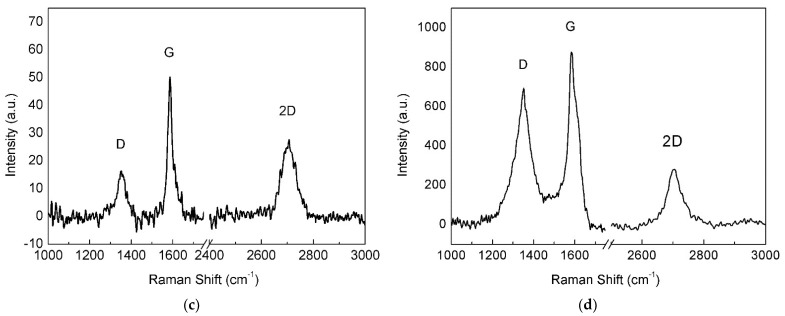
Raman spectra of graphene material grown over the as-plated copper catalyst for different times: (**a**) 2, (**b**) 4, (**c**) 6 and (**d**) 8 min.

Raman spectra on untreated Cu foils showed a luminescence band [29], typical of Cu substrates irradiated with green light, that was removed in order to properly compare the Raman spectra (Figure 4a–d).

Intensity of the 2D band progressively increased with increasing deposition time, and the D band was always present, probably related to the small dimension of the graphene domains. The graphene sample with 8 min of deposition time displayed a Raman spectrum (Figure 4d) with low-intensity 2D band and initial merging of G and D bands, typical of less ordered systems [30]. Thus, Raman analysis demonstrated that the optimal deposition time for growing graphene films on the pristine electroformed Cu substrate was nearly 6 min and the spectral features associated with this treatment time are reported in Table 1. Although the 2D band is relatively intense (I_2D_/I_G_ = 0.6) and can be fitted with a single Lorentzian curve, its large FWHM linewidth (58 cm^−1^) suggests that the film consists of FLG, for the reasons explained above.

**Figure 5:**
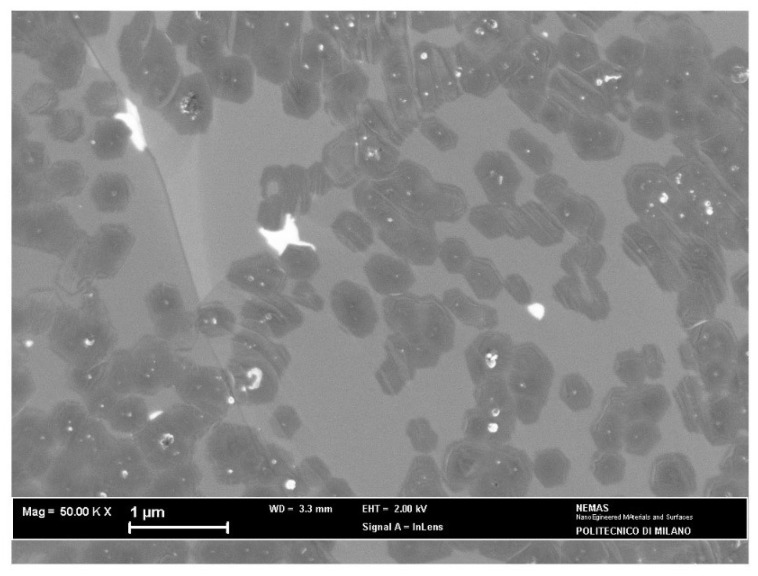
SEM surface image of graphene grown over the as-plated copper catalyst for 6 min.

SEM observations (Figure 5) revealed the existence of many graphene islands growing on the Cu substrate and merging in some areas to form a continuous deposit. According to the Raman analysis, these islands contain more than one graphene layer, showing that self-limiting growth on Cu substrates is not ensured in APCVD processes, in agreement with other results [7,10].

Some isolated flakes exhibit hexagonal shape, which is favored by the etching action of hydrogen (97 vol% in the CVD atmosphere) at the edge sites [10]. The average size of these flakes can be estimated as 0.41 μm with a standard deviation of 0.05 μm.

### 3.2. Annealed Cu Substrate

**Figure 6:**
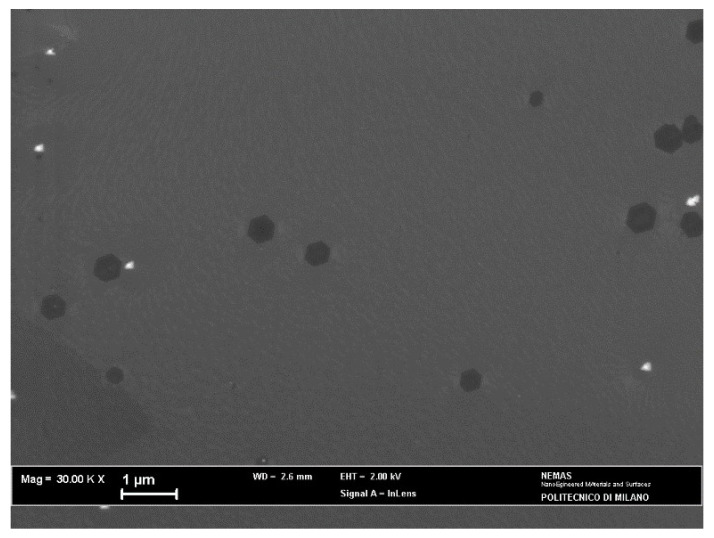
SEM micrograph of the annealed copper catalyst after the APCVD process.

**Figure 7:**
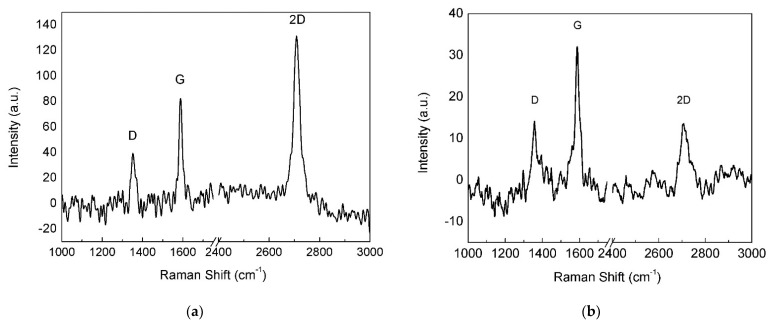
Raman spectra of (**a**) monolayer and (**b**) FLG areas on the annealed Cu substrate.

When the annealed Cu foil was exposed for 2 min to the H_2_/CH_4_ atmosphere, continuous monolayer graphene was found (Figure 6), as revealed by the Raman spectrum (Figure 7a), showing a single intense 2D band. However, nucleation islands with more than one layer could be present (see discussion below). Few-layer/multilayer flakes look darker on SEM images [31] and cover a minor part of the surface in Figure 6, including the dark triangle in the bottom left corner and sporadic hexagonal islands.

### 3.3. Annealed-polished Cu Substrate

**Figure 8:**
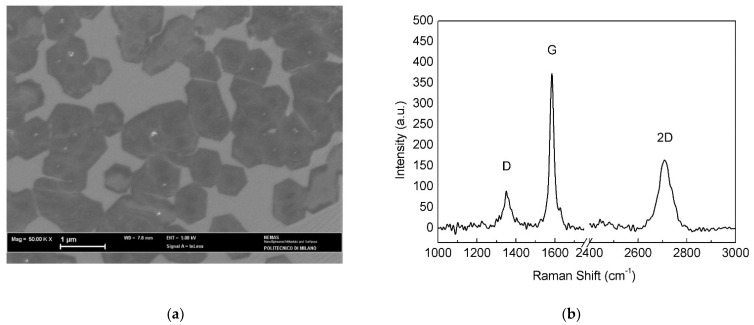
(**a**) SEM micrograph and (**b**) Raman spectrum of the annealed-polished catalyst after the APCVD process.

The annealed-polished catalyst was subjected to longer CVD exposure (20 min) compared to pristine and annealed foils, since shorter treatments (2, 4 and 6 min) led to Raman spectra similar to that in Figure 4a, suggesting that the nucleation density was low and, hence, the deposited material was hardly detectable. After a growth time of 20 min, Raman analysis (Figure 8b) presented good results and SEM observation revealed a 10% increase in surface coverage (Figure 8a) with respect to as-plated Cu. The average size of the graphene islands also increased (0.75 ± 0.03 μm), although only a few isolated hexagonal flakes could be distinguished and used for this estimate.

## 4. Discussion

All the Raman spectra presented show the main peaks typical of graphene. As can be seen from Table 1, the G peak always appears in the range from 1584–1587 cm^−1^, slightly shifted towards higher wavenumbers with respect to pristine graphene. In addition, the position of the 2D peak shifted likewise. On the other side, the D-peak position seems to be consistent in all the reported spectra, remaining within the instrument spectral resolution. Full width at half maximum for the 2D peak is typical of multilayer materials as regards the as-plated and annealed-polished catalysts (>50 cm^−1^), while it was almost half of that value for the SLG grown on the annealed Cu substrate, in good agreement with the literature. Intensity ratios are always lower than 1 in the case of I_D_/I_G_, symptomatic of a good degree of order but small grain size, while the I_2D_/I_G_ ratio varied depending on the number of layers present (see *Results*).

### 4.1. As-Plated Cu Substrate

Raman analysis accomplished on electroformed Cu in the as-plated state revealed the presence of 3 to 4 graphene layers grown on this substrate, as shown by the values of the 2D-band FWHM and I_2D_/I_G_ ratio. The growth route is characterized by the formation of several islands with incomplete substrate coverage (Figure 5) and can be related to the uneven profile of the Cu surface, where many protrusions were noticed in the AFM image (Figure 3a). Indeed, growth of multilayer graphene was observed to take place preferentially at step edges and ridges on the surface of Cu foils [32,33], leading to insufficient density of carbon adatoms on remaining bare Cu for achieving full surface coverage [31,32].

Multilayer islands probably formed by nucleation and growth of new layers next to the substrate [34]. Diffusion of C adatoms under the graphene sheets is permitted by the weak interaction between graphene and Cu substrate and is favored when C adsorption at graphene edges is hindered [34]; this typically occurs when edge sites are terminated by hydrogen in CVD atmospheres with high partial pressure of hydrogen [35], similar to that used in the present work.

### 4.2. Annealed Cu Substrate

After annealing, the Cu substrate acquired almost complete (111) crystalline orientation and its surface became highly smooth, thus, promoting monolayer graphene growth. In fact, the 2D band FWHM is close to the value measured on Si/SiO_2_ for graphene obtained by mechanical cleavage of graphite [19]. Moreover, the I_2D_/I_G_ ratio was high enough to confirm the presence of a single monolayer [35]. The I_D_/I_G_ ratio was higher with this sample, suggesting that small domains exist in monolayer graphene, associated with relatively high nucleation density.

The existence of monolayer graphene on annealed Cu could be related to its strong crystalline orientation and the high growth rate of graphene on Cu (111) planes [22,36]. However, experiments performed in low pressure atmospheres (2 to 30 Torr) showed that single layer graphene can grow even on polycrystalline Cu with random grain orientation [14]. An additional important result of the annealing treatment is the development of a greatly uniform surface (Figure 3b), without a significant number of defects such as marks and step edges, where preferential growth of multilayer graphene can take place [32]. This is a distinctive advantage of the electroformed substrate, considering that rolling marks typical of commercial Cu foils are not eliminated by common annealing treatments [32,33].

The formation of FLG regions on the annealed substrate (Figure 7b) may be due to the high partial pressure of methane in the CVD atmosphere. Synthesis of graphene films on Cu foils at ambient pressure was investigated by changing the methane concentration in Ar-H_2_-CH_4_ gas mixtures [31]. Under a CH_4_ partial pressure of 1 Pa, single layer graphene with rare multilayer domains was produced; the number of these domains increased at higher CH_4_ concentrations. If results coming from low-pressure CVD experiments [3,9,14,33] are included, it is noted that single layer graphene was grown under CH_4_ partial pressures ranging from 1 Pa to 63 Pa. Hence, the high CH_4_ partial pressure (3 kPa) used in this work was expected to produce massive multilayer graphene [9,31]. On the contrary, we found single layer graphene on annealed electroformed Cu, suggesting that the quality of the film grown on this catalyst can be improved by decreasing the methane content in the CVD atmosphere.

It can be noted that a consequence of using high partial pressures of methane is the reduction in the process time, which decreases from 30–60 min for pressures lower than 63 Pa [3,9,14,33] down to a few minutes or less when the pressure is of the order of some kPa, as observed in the present work and in Ar-H_2_-CH_4_ atmospheres [10].

### 4.3. Annealed-Polished Cu Substrate

Electropolishing altered the smoothness of the original annealed surface, creating a number of defects that were regarded as preferential sites for nucleation and growth of multilayer graphene. As a result, the growth mode on the annealed-polished surface was similar to that observed on the as-plated Cu substrate, although the reaction time needed to reach a good surface coverage was longer (20 min compared to 6 min), probably because of the lower concentration of surface defects (Figure 3a,c).

On the other hand, longer deposition time produced the most ordered of all three films, with good surface coverage and larger domains. The 2D band was less shifted but also less intense, as can be seen from the I_2D_/I_G_ ratio; FWHM was quite large; in conclusion, data support the hypothesis of having 4 to 5 graphene layers on top of annealed-polished electroformed Cu substrate.

As reported at the beginning of the Discussion section, all the G-band positions in Table 1 shifted to higher wavenumbers with respect to pristine graphene. This could be attributed to the interaction with the metal substrate, which is always present for CVD samples [6]. In particular, since Cu electronic structure does not induce any hybridization of the graphene grown on its surface, this effect should be due to physisorption. These interactions are known to slightly modify phonons dispersion and, in turn, reduce the Kohn anomalies, exactly as if the graphene was doped. This induces a stiffening of the crystal, which causes the upshift of the G-line position [37]; it also causes a reduction in the electron-phonon coupling (EPC), which attenuates the Raman signal in single layer samples.

## 5. Conclusions

In this paper, we reported the study of graphene/FLG nucleation and growth on fully self-developed Cu substrates made by electroforming process. A complete morphological characterization of the substrates was presented, along with related graphene film Raman analysis. Moreover, electroformed Cu foils were treated to change surface conditions, and modifications in deposited film quality were highlighted. In particular, a conventional annealing treatment produced a highly smooth surface, where single layer graphene was grown. According to the existing literature, massive multilayer graphene would be expected to form in place of single layer graphene, under the investigated growth conditions. Conversely, Cu substrates with uneven surface generated graphene films consisted of few-layer/multilayer islands. Future developments will focus on testing the proposed AP-CVD process on different electroformed substrates, to further explore the electroplating potentialities in graphene synthesis. In addition, the CVD process itself can be improved to yield a desired number of graphene monolayers, mainly by optimizing the methane content in the growth atmosphere.

## Figures and Tables

**Table 1:** Comparison of Raman features for the examined samples.

Sample	Pos(G) (cm^−1^)	Pos(D) (cm^−1^)	Pos(2D) (cm^−1^)	FWHM (2D) (cm^−1^)	I_2D_/I_G_	I_D_/I_G_
Cu as-plated	1585	1353	2707	58	0.6	0.4
Cu annealed	1587	1352	2709	26	1.5	0.5
Cu annealed-polished	1584	1353	2705	58	0.5	0.3
